# Prevalence and factors associated with syphilis in parturient women in Northeast, Brazil

**DOI:** 10.1186/1471-2458-13-206

**Published:** 2013-03-07

**Authors:** Maria Alix Leite Araújo, Silvio Carlos Rocha de Freitas, Heber José de Moura, Ana Paula Soares Gondim, Raimunda Magalhães da Silva

**Affiliations:** 1 Collective Health Program, University of Fortaleza - UNIFOR, Av. Washington Soares, 1321, Edson Queiróz, Fortaleza, Ceará, CEP 60.811-905, Brazil; 2 Municipality of Fortaleza, Ceará, Rua Assunção, 283, Centro, Fortaleza, Ceará, CEP 60.055-090, Brazil; 3 Administration Program, University of Fortaleza – UNIFOR, Av. Washington Soares, 1321, Edson Queiróz, Fortaleza, Ceará, CEP 60.811-905, Brazil; 4 Pharmaceutical Sciences Program, Federal University of Ceará, Rua Capitão Francisco Pedro, 1210, Bairro Rodolfo Teófilo, Fortaleza, Ceará, CEP 60.430-170, Brazil

**Keywords:** Syphilis, Parturient, Prevalence, Associated factors

## Abstract

**Background:**

Congenital syphilis is a major public health concern, even after the implementation of intervention protocols in several countries. This study aimed to analyze the prevalence and socio-demographic, behavioral and institutional factors associated with syphilis in parturient women attending public maternity hospitals in Northeast, Brazil.

**Methods:**

A cross-sectional study was conducted from June to September 2010 with a proportionate stratified sampling of 222 parturient women using a structured questionnaire. The study analyzed socio-demographic, behavioral and institutional variables. The structured questionnaire was conducted with parturient women and complementary information was obtained through hospitals records, admission forms and prenatal cards. Data were stored using the Statistical Package SPSS version 18. A descriptive statistical analysis was performed using frequency distribution, central tendency and measures of spread for the variables. A bivariate analysis was done using chi square test and Fisher’s exact test, with a significance level of 5% and a 95% confidence interval, in order to analyze the relation between the variables and risk factors for syphilis. The multivariate logistic regression analysis was done in the statistical package STATA, version 11.0.

**Results:**

The prevalence of syphilis in parturient women was 7.7%. The bivariate analyses showed that the rate was higher among women who: were from Fortaleza (p = 0.019), studied for less than nine years (p = 0.044), had more than one sexual partner in life (p = 0.021), did not live with partner (p = 0.022), used illegal drugs (p < 0.0001), whose partner used illegal drugs and had diagnosis of syphilis (p = 0.001 and p < 0.0001 respectively). The non-adjusted analysis found significant positive association between syphilis and the following variable: being from Fortaleza (OR = 7.26; CI 95% = 1.49-100.20), having studied for less than nine years (OR = 7.97; CI 95% = 0.87-12.89), having more than one sexual partner in life (OR = 3.75; CI 95% = 1.59-107.11), not living with partner (OR = 3.75; CI95% = 1.03-12.15), and parturient women and partner used illegal drugs (OR = 7.34; CI95% = 1.69-27.57; OR = 4.93; CI95% = 1.58-16.05), respectively. The adjusted multiple logistic regression analysis showed that none of the variables remained significant.

**Conclusion:**

This study enabled to identify a high prevalence of syphilis in parturient women and that this situation is associated with socio-demographic, behavioral and institutional variables.

## Background

The congenital syphilis (CS) is still a major public health concern, even after the implementation of intervention protocols in several countries [1].The worldwide prevalence of syphilis during pregnancy ranges from 0.11 to 8.40 [2].

In Brazil, the global prevalence is 2.6, ranging from 1.0 to 4.4. In Fortaleza city (the capital of the state of Ceara) this prevalence is 2.3 [3]. In all, 29,544 cases of syphilis amongst pregnant women were registered from June 2005 to June 2010 [4]. The analysis of records from the year 2000 to 2010 found that 75.5% of mothers with syphilis received prenatal care and only 55.4% of them were diagnosed during pregnancy [4,5]. In 2009, the detection rate for CS ranged from 0.2 to 5.8 per 1000 live births among Brazilian States [4]. Even considering the health records failures in Brazil, these data show the low quality of prenatal care.

When syphilis in pregnant women is not properly treated, it may cause abortion, neonatal and fetal death, preterm birth, intrauterine growth retardation, and long-term harm to the baby’s health [6,7]. The persistence of the disease punctuates the need for international and national actions for an effective implementation of necessary measures, once its control may contribute to the reduction of infant mortality, which is one of the Millennium Development Goals [8].

Universal screening for syphilis in pregnant women using the Venereal Diseases Research Laboratory (VDRL) and non-treponemal tests along with a high quality prenatal care are some of the most important strategies recommended by the World Health Organization (WHO)[2] and the Brazilian Ministry of Health [9] for the elimination of CS as a public health problem. In Brazil, the national policy recommends three VDRL tests in pregnant women: two during prenatal care and one during the childbirth.

The diagnosis of syphilis can be confirmed through the deployment of treponemal tests. The high cost of such tests and the limited numbers of laboratories either hinder their frequent use in healthcare services or delay their results, hampering the performance of therapeutic and educative interventions to prevent vertical transmission. Thus, the Ministry of Health of Brazil recommends the treatment of all pregnant women with positive VDRL, regardless of the titration, provided there’s no history of previous proper treatment [10].

It is evident that the recommendation for the treatment of positive VDRL cases without a confirmatory test can cause an overtreatment of pregnant women. It is worth pointing out that it is not a specific test, and it also may present false-positive results [11,12]. For that reason, this test would be best recommended for a post-therapeutic follow-up. The ideal procedure would be to ensure a timely confirmatory test.

Concerning the urgent need to ensure prevention and control of CS and considering that healthcare services use the VDRL to detect syphilis and treat pregnant women in order to prevent vertical transmission, the aim of this study was to analyze the prevalence and socio-demographic, behavioral and institutional factors associated with syphilis in parturient women attending public maternity hospitals in Northeast, Brazil. Identifying factors related to syphilis in parturient women may contribute to the development of strategies for prevention and control of syphilis in pregnant women and consequently to control CS.

## Methods

### Type of study and setting

This is a cross-sectional study with parturient women attending seven public maternity hospitals of Fortaleza city, Ceara, Brazil, performed from June to September 2010. These hospitals are part of the Brazilian National Healthcare System (SUS) and offer secondary and tertiary care. They work along with the municipal and state health secretariats and a university.

Fortaleza city has six Regional Executive Boards (REB) and it has around 2 500,000 inhabitants with 888,966 (35.5%) women of reproductive age among them [13]. The healthcare services offered by SUS consist of a hierarchal network of 1712 healthcare units, including public and private services and also blood banks.

### Study populations and sample

The study population consisted of parturient women who were at the hospitals lodging, including all the women who performed the VDRL test at the time of their admission for the delivery. The sample calculation used 22.232 childbirths from women assisted by SUS with prevalence of 2.3% of syphilis in pregnant women in Fortaleza [3], a 2% sampling error and 95% confidence interval. The study used a proportional stratified sample of 222 pregnant women considering the total number of childbirths performed in each maternity hospital in 2009. The formula f = n/N calculated the sampling fraction and n1 = f. N1/N calculated the number of elements that would be observed in each hospital [14].

### Data collection

The structured questionnaire was conducted with the parturient women, and complementary information was obtained through hospitals records, admission forms and prenatal cards. The authors conducted a pilot test using the questionnaire in order to improve the interviewer’s understanding and make any corrections, if necessary.

Undergraduate nursing students were properly trained to visit the hospitals and apply the survey and also obtain complementary information. The VDRL test was done with all parturient women at the time of their admission for the delivery even if it had been done previously during prenatal, following the recommendations of the Ministry of Health [10]. The tests were done in all the hospitals where the childbirth occurred using the flocculation reaction test [15].

### Statistical analysis

The study analyzed the results of serology for VDRL in parturient women and socio-demographic variables: origin, age, schooling, family income, occupation (working or studying), and living with a partner; Behavioral variables: initiation of sexual activity, number of sexual partners in life, living with partner, history of having changed sex for money, use of illegal drugs (marijuana, crack, cocaine); Individual and partner’s risk factors: history of syphilis before pregnancy, occurrence of genital injury, partner diagnosed with syphilis, partner using illegal drugs; Institutional variables: prenatal care (at least one consultation), number of consultations, gestational age at the initiation of prenatal care, VDRL test in prenatal care during the 1^st^ and 3^rd^ trimesters. The participants were asked about any signs or symptoms of genital injury and previous treatment for syphilis.

Syphilis diagnosis at delivery was considered a dependent variable while behavioral, institutional and socio-demographic aspects were considered independent variables.

Analyses were performed using the program SPSS version 18 and STATA version 11.The SPSS was used for the insertion of data and presented descriptive statistical analysis using frequency distribution for central tendency and measures of dispersion and also to split variables in groups. A bivariate analysis was done using Pearson’s Chi-square test and Fischer’s Exact Test, with a significance level of 5% and a 95% confidence interval, in order to analyze the relation between dependent and independent variables and risk factors for syphilis. The multiple logistic regression analysis was done in STATA, and all the covariates were entered simultaneously into the multiple regression models.

### Ethical considerations

The study was approved by the Ethics in Research Committee of two of the participating maternity hospitals according to Protocol N^o.^117/09. The access to information from medical records and admission forms occurred after having the depositary sign a form. The directors of the maternity hospitals signed a document authorizing authors to conduct the research and all parturient women signed the Informed Consent Form.

Aiming to meet the requirements of Informed Consent for research involving adolescents in Brazil [16], the legal representatives of those participants signed the form, keeping the right to the same information.

The parturient women identified with positive VDRL started the treatment with benzathine benzylpenicillin in the maternity hospitals and their partners were summoned and referred to a primary healthcare unit for follow-up care.

## Results

During the period of the study, 222 parturient women participated in the research (30 were excluded, 23 who were admitted due to abortion, five who did not take the VDRL test at their admission and two who refused to sign the Informed Consent Form).

In all, 173 (77,9%) women were older than 19 years old, 120 (54.1%) have studied for less than nine years and 177 (83.5) had a family income of less than two minimum wages. The study showed that 16 women (7.2%) used some kind of non-injectable illegal drugs (marijuana and/or cocaine and/or crack cocaine), 56 (25.2%) said their partners used the same kind of illegal drugs and 19 (8.6%) had previous genital complaints. It is worth saying that none of the respondents reported using injectable drugs or its use by their partners (Table 1).

**Table 1:** Socio-demographic and behavioral factors associated with syphilis in parturient women admitted to public maternity hospitals

**Variables**	**Frequency**	**Syphilis**	**p value**
	**n (%)**	**n (%)**	
Origin			0.019
Fortaleza	157 (70.7)	16 (10.2)	
Countryside	65 (29.3)	01 (1.5)	
Age (years)			0.459
≤ 19	49 (22.1)	03 (6.1)	
> 19	173 (77.9)	14 (8.1)	
Schooling (completed years of study)			0.044
≤ 9	120 (54.1)	13 (10.8)	
> 9	102 (45.9)	04 (3.9)	
Family income (minimum wages)*			0.220
≤ 2	177 (83.5)	11 (6.2)	
> 2	35 (16.5)	04 (11.4)	
Occupation (working/studying)			0.956
Yes	90 (40.5)	07 (7.8)	
No	132 (59.9)	10 (7.6)	
Initiation of sexual activity (years)			0.110
≤ 19	196 (88,3)	17 (8.7)	
> 19	26 (11.7)	-	
N^o.^ of sexual partners in life			0.021
1	68 (30.6)	01 (1.5)	
> 1	154 (69.4)	16 (10.4)	
Living with partner			0.022
Yes	190 (85.6)	11 (5.8)	
No	32 (14.4)	06 (18.8)	
Use illegal drugs			<0.0001
Yes	16 (7.2)	05 (31.3)	
No	206 (92.8)	12 (5.8)	
Partner uses illegal drugs			0.001
Yes	56 (25.2)	10 (17.9)	
No/Ignored	166 (74.8)	07 (4.2)	
Partner had diagnosis of syphilis			<0.0001
Yes	12 (5.4)	10 (58.8)	
No/Doesn’t know	210 (94.6)	07 (3.3)	
Changed sex for money			0.145
Yes	09 (4.1)	02 (22.2)	
No	213 (95.9)	15 (7.0)	

Fortaleza, Ceará, Brazil, 2010.

*Current minimum wage $ 251.281.

*10 people did not respond.

A total of 17 women (7.7%) had a positive VDRL, and among them, 12 (70.6%) already knew the result during prenatal care. Ten women (83.3%) reported receiving at least one dose of benzathine benzylpenicillin (2.400.000 UI) and no one could say the total of doses administered. It is worth saying that 97 women (43.7%) initiated sexual life at the age of 15 years or less and among them, 13/17 (13.4%) had a positive VDRL in childbirth (82.3% of the positive cases).

The rate was higher among women who: were from Fortaleza city (p = 0.019), studied for less than nine years (p = 0.044), had more than one sexual partner in life (p = 0.021), did not live with partner (p = 0.022), used illegal drugs such as marijuana, cocaine and crack (p < 0.0001) and whose partner used illegal drugs and had diagnosis of syphilis (p = 0.001 and p < 0.0001, respectively).

Table 2 shows the analysis of institutional factors associated with syphilis. A total of 210 women (94.6%) had at least one prenatal consultation and 179 (85.2%) performed the consultation in primary healthcare. Among the ones who received prenatal care, 206 (98.1%) took at least one VDRL test. Among them, 49 (23.8%) took it during the first trimester of pregnancy and 86 (41.7%) in the third trimester while 71 (34.5%) took it in the first and third trimesters and four (1.9%) did not take any VDRL tests. Significant association was found in women who did not receive prenatal care (p < 0.001) and did not take VDRL during prenatal care (p < 0.001) with the occurrence of positive VDRL.

**Table 2:** Analysis of institutional factors associated with syphilis in parturient women admitted to public maternity hospitals

**Variables**	**Parturients**	**Syphilis**	**p value**
	**n (%)**	**n (%)**	
Received prenatal care			<0.001
Yes	210 (94.6)	12 (5.7)	
No	12 (5.4)	05 (41.7)	
No. of prenatal consultations*			0.228
≤ 6	127 (60.5)	09 (7.1)	
> 6	83(39.5)	03 (3.6)	
Initiation of prenatal (weeks)*			0.132
< 12	75 (35.7)	02 (2.7)	
≥ 12	135(64.3)	10 (7.4)	
VDRL during prenatal (at least one)*			<0.001
Yes	206 (98.1)	08 (3.9)	
No	04 (1.9)	04 (100.0)	
VDRL in the 1^st^and 3^rd^trimesters*			
Yes	73(34.8)	04 (5.5)	0.592
No	137 (65.2)	08 (5.8)	

Fortaleza – Ceará, Brazil, 2010.

*Considered only who received prenatal care.

The non-adjusted analysis found a significant positive association between syphilis and the following variables: being from Fortaleza (OR = 7.26; CI 95% = 1.49-100.20), having studied for less than 9 years (OR = 7.97; CI 95% = 0.87-12.89), having more than one sexual partner in life (OR = 3.75; CI 95% = 1.59-107.11), not living with the partner (OR = 3.75; CI95% = 1.03-12.15) and use of illegal drugs by parturient women and partner (OR = 7.34; CI95% = 1.69-27.57; OR = 4.93; CI95% = 1.58-16.05), respectively. The adjusted multiple logistic regression analysis showed that none of the variables remained significant (Table 3).

**Table 3:** Adjusted analysis of variables: origin, years of schooling, number of sexual partner, living with the partner, use illegal drugs, partner uses illegal drugs, associated with syphilis in parturient women admitted to public maternity hospitals

**Variables**	**Syphilis**		
	**Adjusted odds**	**CI 95%**	**p**
Origin from Fortaleza	4.70	0.57-38.37	0.148
Less than 9 years of schooling	2.04	0.59-7.09	0.258
More than one sexual partner in life	4.26	0.51-35.58	0.181
Not living with the partner	2.41	0.74-7.84	0.143
Use illegal drugs	2.39	0.59-9.59	0.217
Partner uses ilegal drugs	1.70	0.507-5.75	0.388

Fortaleza, Ceará, Brazil, 2010.

## Discussion

The 7.7% prevalence of syphilis in parturient women was high when compared to another study conducted in Bolivia [17]. VDRL test is used in healthcare routine due to the difficulty in access to confirmatory tests. Furthermore, it has a high sensitivity, low cost and a fast treatment response [10]. In Brazil, it is recommended the performance of three tests in pregnant women: two during prenatal and one in childbirth.

Regarding factors associated with positive VDRL, it was found a positive association with the use of illegal drugs. A study conducted with parturient women in Vitoria city, Espirito Santo, did not find a positive association with these variables in the multiple regression design [18]. Other studies showed that low socioeconomic level, use of drugs (crack and cocaine), lack of prenatal care and STD symptoms are found to be factors associated with syphilis [19,20].

The high rate of positive VDRL cases among women from Fortaleza city was expected, considering that the care for pregnant women from other cities usually occurs due to the need for a more qualified care that is available in reference maternity hospitals.

Most of the parturient women had their first sexual relation when they were less than 15 years of age and all women with positive VDRL initiated their sexual activities when they were 19 or younger. This shows that the early initiation of sexual activity may favor the exposure to STD [21]. It is necessary to improve healthcare services in order to expand and prioritize its access by the young population. These findings show the importance of intensifying the health policy for adolescents with more actions against STD and precocious pregnancy, which are markers of vulnerability in this age group.

It is true that, in Brazil, the initiation of sexual activity occurs earlier [21-24] and that it exposes youngsters to STD due to the unawareness of the vulnerability conditions, changing of sexual partners because of an unstable and immature relationship and also the non-use of condoms, especially when they consider trusting the partner.

A relevant percentage of women who were diagnosed with positive VDRL during prenatal reported receiving at least one dose of benzathine benzypenicillin. However, they did not know how many doses were administered, highlighting the low quality of health care that cannot be assessed considering only the number of consultations performed.

The quality of health care spans the valorization of pregnant women’s counseling, especially of those who have diseases which the transmission to the baby can be avoided, such as syphilis and HIV. A study conducted in the city of Rio de Janeiro with pregnant women during prenatal care in public health services showed that 15% of them did not know whether they had done VDRL or not, demonstrating flaws in the guidelines during prenatal care [25]. In general, health professionals value this action during pregnancy when it concerns to HIV but not to syphilis [25,26].

However the findings of this study need to be interpreted with caution since it considered the results of VDRL test that does not establish the syphilis diagnosis. It recognizes the need to conduct a confirmatory treponemal test, but the objective of this study was to analyze the results of VDRL considering that the confirmatory test is not available in routine of the health services in Fortaleza. Therefore, the Ministry of Health recommends that pregnant women with positive VDRL test must be treated for syphilis regardless of the titration.

This study draws attention to the number of parturient women that did not have any VDRL tests during prenatal, confirming the findings of another study conducted in Brazil about failures in health services regarding the adoption of measures to prevent and control vertical transmission of syphilis. A study showed that the early capture and reception of pregnant women, as well as the correct and timely detection and treatment for syphilis, especially in their sexual partners, is still a challenge for the control of congenital syphilis [27].

It is worth noting that non-injectable drug use (marijuana, cocaine and crack) was associated with positivity for VDRL. This situation calls attention to the importance of considering the use of this kind of drugs in sexually transmitted diseases.

## Conclusion

This study enabled to identify a high prevalence of syphilis in parturient women in Fortaleza city, Northeastern Brazil, and that this situation is associated with socio-demographic, behavioural and institutional variables.

## Competing interest

We recognize that this manuscript does not present financial conflicts among the authors. We certify that this manuscript was submitted only and exclusively to the BMC Public Health.

## Authors’ contribution

MALA – developed the project, analyzed and interpreted data, and helped in the final review of the article. SCRdF – developed the project, collected, analyzed and interpreted data. HJdM – performed the statistical treatment, analyzed data and helped in the final review of the article. APSG – analyzed and interpreted data and helped in the final review of the article. RMdS – analyzed and interpreted data and helped in the final review of the article. All authors read and approved the final manuscript.

## Authors’ information

Silvio Carlos Rocha de Freitas, MS, Secretary of the Municipality of Fortaleza, Ceará.

Maria Alix Leite Araujo, PhD, Professor, University of Fortaleza.

Heber José de Moura, PhD, Professor, University of Fortaleza.

Ana Paula Soares Gondim, PhD, Professor, University of Fortaleza.

Raimunda Magalhães da Silva, PhD, Professor, University of Fortaleza.

## Pre-publication history

The pre-publication history for this paper can be accessed here:

http://www.biomedcentral.com/1471-2458/13/206/prepub
